# Analysis of Magnitude and Direction of Body Composition Asymmetries in Male Brazilian Jiu-Jitsu Athletes: Pilot Study

**DOI:** 10.3390/sports13020054

**Published:** 2025-02-12

**Authors:** Alex Ojeda-Aravena, Rafael L. Kons, Jairo Azócar-Gallardo, Xurxo Dopico-Calvo, Aida Fernández, Marcelo Tuesta-Roa, Mauricio Cresp-Barria, Jorge Olivares-Arancibia, Eduardo Báez-San Martín

**Affiliations:** 1 Dirección de Docencia, Universidad de Los Lagos, Osorno 1305, Chile; 2 Department of Physical Education, Faculty of Education, Federal University of Bahia, Salvador 40170-110, BA, Brazil; rafael.kons@ufba.br; 3 Departamento de Ciencias de la Actividad Física, Universidad de Los Lagos, Osorno 1305, Chile; jairo.azocar@ulagos.cl; 4 Programa de Investigación en Deporte, Sociedad y Buen Vivir (DSBv), Universidad de Los Lagos, Osorno 1305, Chile; 5 Performance and Health Group, Department of Physical Education and Sport, Universidade da Coruña, 15001 A Coruña, Spain; xurxo.dopico@udc.es; 6 Faculty of Education, Universidad Central de Chile, Santiago 8370292, Chile; aida.fernandez@ucentral.cl; 7 Exercise and Rehabilitation Sciences Laboratory, School of Physiotherapy, Faculty of Rehabilitation Sciences, Universidad Andres Bello, Santiago 8370292, Chile; marcelo.tuesta@unab.cl; 8 Department of Innovation and Education, Faculty of Education, Catholic University of Temuco Chile, Temuco 4810399, Chile; mcresp@uct.cl; 9 AFySE Group, Research in Physical Activity and School Health, School of Physical Education, Faculty of Education, Universidad de las Américas, Santiago 8370292, Chile; jolivares@udla.cl; 10 Carrera de Entrenador Deportivo, Escuela de Educación, Universidad Viña del Mar, Viña del Mar 2580022, Chile; eduardo.baez@upla.cl; 11 Laboratorio de Evaluación y Prescripción de Ejercicio, Facultad de Ciencias de la Actividad Física y del Deporte, Universidad de Playa Ancha, Valparaíso 850, Chile

**Keywords:** asymmetry, Brazilian Jiu-Jitsu, sports sciences, body composition

## Abstract

In order to optimize body composition and its components, it is of interest to analyze inter-limb asymmetries in athletes of popular combat sports such as Brazilian Jiu-Jitsu (BJJ). This study aimed to assess the magnitude and direction of body composition asymmetry in competitive male BJJ athletes. Seventeen experienced and competitive male BJJ athletes (age 34.59 ± 8.00 years, 172.94 ± 5.46 cm, BJJ experience 7.88 ± 5.57 years, and 3.71 ± 1.05 days of weekly volume training), including Guard Fighters (*n* = 7) and Pass Fighters (n = 10), participated in this study. In a single session, whole-body and segmental upper limb and lower limb body composition (BC) was assessed utilizing dual-energy X-ray absorptiometry (DXA). The assessment included total mass (TM), fat mass (FM), fat mass percentage (%FM), fat-free mass (FFM), and bone mineral content (BMC). Absolute differences between limbs were analyzed using a paired *t*-test. A comparison of BC asymmetries according to combat styles was conducted using an independent *t*-test. The effect size (ES) was interpreted as Cohen’s d. The main results revealed significant asymmetries in the upper limbs (ULs) of the total group; greater values were found on the left side for TM (*p* = 0.009, ES = 0.725), FM (*p* = 0.016, ES = 0.650), FFM (*p* = 0.026, ES = 0.594), and BMC (*p* < 0.001, ES = 0.993). In Pass Fighters, differences favored the right side in TM (*p* = 0.003, ES = 1.277), FM (*p* = 0.009, ES = 1.039), FFM (*p* = 0.011, ES = 1.000), and BMC (*p* < 0.001, ES = 1.916). In contrast, Guard Fighters showed no discrepancies in these parameters. No notable disparities were observed in the lower limbs. This pilot study reveals that BJJ athletes present significant asymmetries in BC, particularly in the ULs, with a marked predominance on the right side, especially in Pass Fighters.

## 1. Introduction

Brazilian Jiu-Jitsu (BJJ) is a modern martial art and combat sport that focuses on subduing the opponent through submission and joint-lock techniques. BJJ athletes specialize in various fighting styles. Notably, there are Guard Fighters, who prefer to fight from the guard position on the ground, and Pass Fighters, who aim to overcome the guard to achieve a dominant position over their opponent [1]. It is important to note that Guard Fighters and Pass Fighters share several physical traits, such as flexibility, balance, isometric handgrip strength, endurance, and quadriceps and hamstring strength and the ability to perform well in specific BJJ physical tests [2]. However, Pass Fighters tend to excel in isometric trunk extension endurance, which can be attributed to the specific demands of their fighting style [2]. BJJ competitions are organized not only by weight categories but also by technical skill levels (grades or belts), which can lead to matchups between competitors of different weights and technical proficiency.

In this context, body composition (BC) is a critical factor in athletic performance and injury prevention in combat sports such as BJJ [3]. The optimal distribution of muscle mass, bone mass, and fat mass significantly influences an athlete’s ability to execute effective techniques, maintain endurance during matches, and comply with weight categories [4]. Additionally, muscle and bone mass play fundamental roles in force production and transmission, whereas excess fat mass can negatively affect physical performance in combat sport athletes [3,4].

Specifically, bilateral asymmetries (i.e., differences in athletic performance and function between the two limbs) have emerged as a research topic, primarily derived from unilateral or bilateral strength tasks. The focus of this topic is on understanding how such discrepancies between limbs can influence performance and injury risk [5,6], as well as being a manifestation of fatigue [7]. Various equations are currently being discussed for determining asymmetries based on unilateral and bilateral tasks, although there is currently no consensus [8,9]. In sports such as judo, bilateral asymmetries have been observed to increase following the execution of sport-specific tests [10]. Meanwhile, in simulated combat in this sport, bilateral asymmetries do not appear to be significant to performance [10].

From this line of research, the analysis of body composition asymmetries (BC-ASs) has been extrapolated by comparing both limbs using advanced technologies including dual-energy X-ray absorptiometry (DXA) [11,12,13,14]. These asymmetries can affect an athlete’s functionality and balance, thereby influencing their technical performance and increasing the risk of injuries [15,16,17]. However, most studies to date have only quantified the magnitude of BC-AS (quantification of the percentage difference between limbs), while the analysis of direction (i.e., which side of the body is less developed) is still in its early stages [18].

In BJJ, the preference for a certain laterality during combat movements can lead to predominant unilateral development, exacerbating BC-AS [19]. Therefore, it is reasonable to suggest that BJJ athletes may present BC-AS. Consequently, it is important to understand that asymmetries in body composition can have a negative impact on physical performance given their relationship to muscle strength and the biomechanics of movement in this sport. Despite the importance of this research topic, there is limited information regarding studies related to the prevalence, magnitude, and direction of BC-AS in BJJ athletes. An individualized approach is essential to accurately and meaningfully assess asymmetries as it allows training and injury prevention programs to be adapted to the specific needs of each athlete. These findings provide valuable information for optimizing training programs, preventing injuries, and improving performance. Coaches and sports professionals can use this information to design specific interventions that address asymmetries and enhance the physical capabilities of athletes by considering their fighting styles and skill levels.

Therefore, this study aimed to assess the magnitude and direction of body composition asymmetry in competitive male BJJ athletes.

## 2. Materials and Methods

### 2.1. Participants

Seventeen male BJJ athletes (age 34.59 ± 8.00 years, 172.94 ± 5.46 cm, BJJ experience 7.88 ± 5.57 years, and 3.71 ± 1.05 days of weekly volume training) participated in this pilot study. Seven athletes were self-declared Guard Fighters and ten Pass Fighters. Ten athletes had black belts, while seven had blue- or purple-level belts. The inclusion criteria were (i) training a minimum of three times per week; (ii) having at least two years of continuous BJJ training; (iii) possessing at least a blue belt; (iv) competing regularly during the last two years; (v) participation in at least one annual International Brazilian Jiu-Jitsu Federation (IBJJF) competition; and (vi) no injuries or health conditions that could affect their performance in this study. The research methodology adhered to the ethical principles of the Declaration of Helsinki and was approved by the local Ethics Committee (CODE: BIOPUCV-H 520-2022). The procedures, benefits of participation, and risks associated with X-ray exposure were communicated one week prior to the assessments.

### 2.2. Measures and Procedures

#### Anthropometric and Body Composition Measures

The athletes participated in two assessment sessions during a one-day period, conducted in an exercise and sports science laboratory maintained at a temperature of 21 °C. DXA-certified professionals measured the anthropometric and BC variables. Athlete height (±0.1 cm) was measured using a stadiometer (Seca 217; Hamburg, Germany) following standard protocols. Subsequently, BC was evaluated using DXA (General Electric Lunar iDXA, Boston, MA, USA). Briefly, the athletes removed any metal or jewelry and laid supine on a scanning table. A whole-body scan was performed in standard mode, following the manufacturer’s recommended procedures. The standard thickness mode was determined by using an automatic scanning function. The results were analyzed using GE EnCORE 2015 v18 software (GE Healthcare) [14]. Each athlete’s race/ethnicity was selected from available software options to accurately reflect their ancestry. Quality assurance was maintained through daily calibrations performed before all scans, using a calibration block provided by the manufacturer. All DXA measurements were performed by a laboratory technician certified in X-ray procedures by the Ministry of Health. Standardized athlete positioning procedures were employed [20]. The BC results included total mass (TM), fat mass (FM), percentage of fat mass (%FM), fat-free mass (FFM), and bone mineral content (BMC) for the total body, upper limbs (ULs), and lower limbs (LLs).

### 2.3. Statistical Analysis

Data were entered in Microsoft Excel and subsequently analyzed using the statistical software JASP version 0.17.3 (JASP Team, 2023, Amsterdam, The Netherlands). Continuous BC descriptors are presented as the mean ± standard deviation with a 95% confidence interval (CI95%). Categorical data were expressed as absolute and relative frequencies in percentage terms. Initially, the assumptions of normality were assessed using the Shapiro–Wilk test and homoscedasticity using the Levene test. The magnitude of asymmetry was described as the percentage difference between the upper and lower limbs, separately based on the higher or lower score obtained, using the equation (([Strongest − Weakest]/Strongest) × 100) [21] modified to higher and lower values according to Bishop et al. [22]. To represent the direction of asymmetries between individual limbs, an IF function was added to Microsoft Excel at the end of the formula *IF(left < right, 1, −1) without altering the magnitude [18]. The mean differences between body segments were determined using a paired sample *t*-test. Differences in the proportions of BC-AS were analyzed using a contingency table with Fisher’s exact test and the Odds Ratio (OR) [23]. Asymmetries based on fighting style were evaluated using an independent sample *t*-test. The magnitude of differences or effect size (ES) was interpreted using Cohen’s d, where values of 0.2, 0.5, and 0.8 indicate small, medium, and large effects, respectively. A *p* value of <0.05 was established as the significance threshold.

## 3. Results

In the normality analysis using the Shapiro–Wilk test, the evaluated variables did not show significant deviations from a normal distribution. In the homogeneity of variance analysis using the Levene test, none of the evaluated variables showed significant differences between the groups (*p* > 0.05).

### 3.1. Global Analysis of Differences and Asymmetries

**Figure 1:**
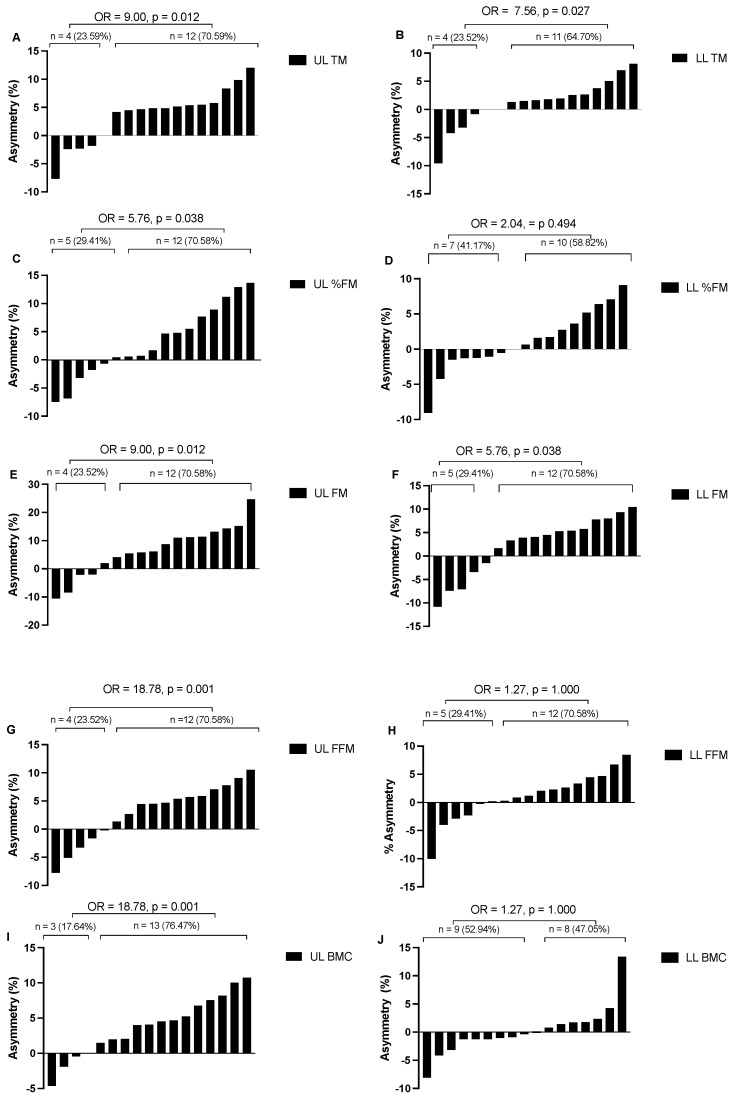
This figure shows the magnitude and direction of the asymmetry percentages in the body composition asymmetries of the athletes. (**A**): UL TM (kg); (**B**): LL TM (kg); (**C**): UL%FM; (**D**): LL FM%; (**E**): UL FM (kg); (**F**): LL FM; (**G**): UL FFM (kg); (**H**): LL FFM (kg) (**I**): UL BMC (kg); (**J**): LL BMC (kg).

Table 1 and Figure 1 provide a detailed description of the differences and asymmetries observed in the BC of the analyzed BJJ athletes. Regarding TM in the ULs, statistically significant differences were observed between the right and left limbs (*p* = 0.009, ES = 0.725). A total of 70.59% of participants showed greater TM in the right limb and 23.53% in the left limb, and 5.88% presented symmetry favoring the right side (OR = 9.0, *p* = 0.012). Conversely, in the LLs, no significant differences were found (*p* = 0.239), although 64.71% of athletes exhibited greater FFM in the right leg and 23.53% in the left leg, and 11.76% showed symmetry, slightly favoring the right side (OR = 7.56, *p* = 0.027).

Concerning %FM, no significant differences were found in the ULs, although 70.59% exhibited a higher %FM in the right limb (OR = 5.76, *p* = 0.038) compared to 29.41% in the left limb. Similarly, no significant differences were observed in the LLs (*p* = 0.872, ES = 0.040), with 58.82% favoring the right leg and 41.18% favoring the left leg (OR = 2.04, *p* = 0.494).

FM in the ULs showed a significant difference (*p* = 0.016, ES = 0.650), with 70.59% of participants presenting a greater FM in the right limb and 23.53% in the left limb and 5.88% showing symmetry (OR = 9.0, *p* = 0.012). In the LLs, no significant differences were found (*p* = 0.131, ES = 0.386), although 70.59% of athletes presented a greater FM in the right leg and 29.41% in the left leg (OR = 5.76, *p* = 0.038).

FFM in the ULs revealed a significant difference (*p* = 0.026, ES = 0.594), with 76.47% of participants presenting a greater FFM in the right limb (OR = 18.78, *p* = 0.001) and 17.65% in the left limb and 5.88% showing no asymmetry. In the LLs, no statistical differences were observed (*p* = 0.296, ES = 0.262), with a relatively balanced distribution of asymmetries between the right leg (52.94%) and the left leg (47.06%; OR = 1.27, *p* = 1.000).

Lastly, BMC in the ULs showed significant differences (*p* < 0.001, ES = 0.993), with 76.47% of athletes presenting a greater BMC in the right limb (OR = 18.78, *p* = 0.001) and 17.65% in the left limb and 5.88% showing symmetry. In the LLs, no significant differences were observed (*p* = 0.872, ES = 0.040), with asymmetry proportions evenly distributed between the right leg (52.94%) and the left leg (47.06%; OR = 1.27, *p* = 1.000).

### 3.2. Comparative Analysis by Fighting Style

Table 2 and Table 3 present a detailed comparison of body composition asymmetries by fighting style (i.e., Guard Fighters vs. Pass Fighters). Regarding TM, the ULs showed similar mean values between Guard Fighters (5.339%) and Pass Fighters (5.164%), without significant differences (*p* = 0.909, ES = 0.057). For the LLs, Guard Fighters presented a higher mean value (4.262%) compared to Pass Fighters (2.518%), although this difference was not significant (*p* = 0.211, ES = 0.644), suggesting a possible trend warranting further analysis. In absolute terms, no significant differences were reported in Guard Fighters for either limb, whereas in Pass Fighters, significant differences were observed in the ULs (*p* = 0.003, ES = 1.277).

Regarding %FM, global asymmetries in the ULs were similar between Guard Fighters (5.965%) and Pass Fighters (5.149%), with no significant differences (*p* = 0.718, ES = 0.181). In the LLs, Guard Fighters also presented slightly higher values (3.961%) compared to Pass Fighters (2.915%), though without statistical significance (*p* = 0.495, ES = 0.345). In absolute terms, no significant differences were reported by fighting style for either limb.

In terms of FM, Guard Fighters recorded a higher mean percentage of asymmetry in the ULs (9.974%) compared to Pass Fighters (8.655%), though this difference was not significant (*p* = 0.661). In the LLs, Guard Fighters presented higher average values (7.434%) compared to Pass Fighters (4.759%), without significant results (*p* = 0.052) and a large effect size (ES = 1.041). In absolute terms, significant differences were reported only in Pass Fighters for the ULs (0.862 kg vs. 0.799 kg; *p* = 0.009, ES = 1.039) and the LLs (2.318 kg vs. 2.230 kg; *p* = 0.011, ES = 1.016).

For FFM, Guard Fighters presented a mean asymmetry percentage in the ULs (5.141%) similar to that of Pass Fighters (5.084%), with no significant differences (*p* = 0.909, ES = 0.057). In the LLs, Guard Fighters exhibited a higher average percentage (4.771%) compared to Pass Fighters (2.300%), though this difference was not statistically significant (*p* = 0.211, ES = 0.644). In absolute terms, Pass Fighters presented significant differences in the ULs (4.138 kg vs. 3.970 kg; *p* = 0.011, ES = 1.000).

Finally, BMC in the ULs showed a lower mean value in Guard Fighters (3.674%) compared to Pass Fighters (5.257%), though the difference was not significant (*p* = 0.330), with a moderate negative effect size (ES = −0.496). In the LLs, differences between both styles were minimal (*p* = 0.748, ES = 0.162). In absolute terms, significant differences were reported only in Pass Fighters for the ULs (0.260 kg vs. 0.247 kg; *p* < 0.001, ES = 1.916).

## 4. Discussion

This study is the first to explore the prevalence, magnitude, and direction of BC-AS in BJJ athletes. To date, most studies have focused on analyzing asymmetries from a functional perspective using bilateral or unilateral strength tasks. However, BC-AS has emerged as an area of study that focuses on understanding the morphological impact of BJJ athlete characteristics. Our main results showed significant differences between both limbs for the ULs in TM, FFM, and BMC, with ES ranging from moderate to large (ES = 0.594 to 1.19). BC-AS could originate from the specific demands and unilateral movement patterns inherent to BJJ, where athletes often use one side of the body more frequently to execute grip, submission, and opponent control techniques.

The predominance towards the right side suggests functional laterality, possibly related to hand dominance and specific BJJ techniques that favor one arm over the other. This finding contrasts with those of other studies in similar sports. For example, Mala et al. [24] observed less pronounced asymmetries in FFM in judokas than in sports that emphasize unilateral limb use, such as fencing and karate, in which athletes showed significant differences between dominant and non-dominant limbs in the arms (*t* = −4.24, *p* < 0.01) and legs (*t* = −6.07, *p* < 0.01). Greater muscle and bone mass on the dominant side could provide advantages in strength and endurance, but it is also proposed that they could generate musculoskeletal imbalances, increase susceptibility to overuse or compensatory injuries on the less developed side, and potentially influence sports performance, although the results to date are inconclusive [17,25].

In the LLs, differences were not statistically significant in most parameters, except in the TM, where the proportions of asymmetry favored the right side (OR = 7.56, *p* = 0.027). The lower magnitude of asymmetries in the LLs could be due to BJJ techniques requiring a more balanced involvement of both legs for movements, such as sweeps, leverages, and displacements. Interestingly, although this study did not directly evaluate functional asymmetries, research in similar sports, such as judo, has reported increases in asymmetries after simulated combats. Kons et al. [10] analyzed the effects of successive judo combats on inter-limb asymmetries and bilateral deficit, finding that the magnitude of asymmetry in unilateral countermovement jump height increased after the second combat (*p* = 0.001).

When considering fighting style, a tendency was observed in the FM of the LLs between Guard Fighters and Pass Fighters (*t* = 2.113, *p* = 0.052, ES = 1.041). Guard Fighters who prefer to fight from the guard position on the ground and extensively use their legs to control and manipulate the opponent might develop greater asymmetries in the fat mass and FFM of the LLs. These differences may be related to the technical and tactical characteristics of each style. Báez et al. [1] observed that Guard Fighters presented a higher percentage of fat mass compared to Pass Fighters, which aligns with our findings. However, Pass Fighters tend to excel in isometric trunk extension endurance, which can be attributed to the specific demands of their fighting style [2].

This highlights the potential differences in morphological terms between fighting styles, which deserves further study. The limitations of this study include the sample size, the absence of female participants, the varying levels of practice among the athletes in the sample, and the lack of consideration of athlete laterality (dominance). These limitations restrict the generalization of the results. Future studies should aim to increase the sample size, include female athletes, and incorporate the assessment of laterality to provide a more comprehensive understanding of asymmetries in sports performance.

Although our study focused on body composition asymmetries, we acknowledge that other factors such as neuromuscular coordination, specific sport techniques, and fatigue also play crucial roles in sports performance and the manifestation of asymmetries. Moreover, while laterality was not directly evaluated in this study, it is a recognized factor that can significantly influence the observed asymmetries. Dominance can affect strength, coordination, and movement efficiency, thereby impacting both performance and injury risk. Therefore, future research should include the evaluation of athlete laterality to better understand its impact on asymmetries and overall performance.

Regardless of the above, coaches, strength professionals, and conditioning professionals should consider BC-AS when designing training programs. This includes incorporating exercises that promote muscular and functional balance between both sides of the body. The inclusion of unilateral training and specific strength exercises could help correct these imbalances and improve performance [5,6]. Additionally, the regular monitoring of body composition and asymmetries can serve as a tool for adjusting training and rehabilitation programs. Ojeda-Aravena et al. [14] highlighted the importance of relating body composition asymmetries to specific performances such as taekwondo, suggesting that individualized assessments can contribute to the optimal development of athletes.

Several coaches emphasize the importance of specific training for increasing sports performance, highlighting its role in metabolic and neuromuscular improvement. Hence, specific training and the repetition of technical gestures and combat actions often create bilateral deficits by working more on the dominant side. While specificity is crucial for enhancing sports performance, it can also lead to more pronounced bilateral deficits, characteristic of high-performance athletes.

In a study by Turnes et al. [26], it was concluded that bilateral symmetry was not associated with better performance in a handgrip strength test carried out with a sample of judokas. On the other hand, when athletes were categorized based on the years of training experience specific to judo, those with the greatest lateral deficit were those who performed more hours of specific training, suggesting that practice and training time may contribute to the deficit. This finding was verified in both studies. However, this deficit should not increase to a level that compromises the athlete’s sporting performance, injury risk, or health, highlighting the importance of studying asymmetry in the upper and lower limbs.

However, a systematic review on the relationship between asymmetry and injury risk by Helme et al. [27] concluded that the evidence supporting the functional asymmetry of the lower limbs as a risk factor for sports injuries was of moderate to low quality. Despite evidence showing that some measures were statistically associated with injury risk in different sporting populations, this field of research is still limited by a high degree of variation in methodological approaches and quality. Researchers should be mindful of these observations before implementing athletic interventions. From this review, it was identified that more research is needed that adopts key recommendations specifically addressing standardized injury definitions, the quantification of asymmetry, and the adoption of a multivariate approach, encapsulating exposure among other variables, within a sample of sufficient size. Without this investigation, no clear results are apparent for accepting or rejecting upper or lower limb functional asymmetry as a risk to sports participation.

Consequently, the topic of BC-AS and its impact on performance, injury risk, and fatigue holds significant potential for further study. This is based on their relevance to the structural morphology and force production of different body limbs. Future research should focus on analyzing the longitudinal effects of BC-AS through strength programs and/or specific BJJ training. Additionally, the efficacy of training programs designed to reduce asymmetries and their impact on performance and injury prevention should be investigated. Furthermore, structural asymmetries could be related to functional measures such as strength, power, and balance to better understand their influence on sports performance. Importantly, future studies should also consider the laterality of athletes, as dominance can significantly influence asymmetries. Including female athletes and athletes with different skill levels would broaden the generalizability of the findings.

## 5. Conclusions

BJJ athletes exhibited significant asymmetries in body composition, especially in the ULs, with a predominance towards the right side. Guard Fighters showed greater asymmetries in the LLs, possibly because of the increased use of the legs in their fighting style. These asymmetries appear to be influenced by the specific demands of the sport and the fighting style.

## Figures and Tables

**Table 1:** Inter-limb differences and body composition asymmetries in BJJ athletes analyzed (*n* = 17).

BC Variables	LIMBS	SIDE	Mean	SD	BC-AS ± SD	*t*	*p*	ES
TM (kg)	ULs	L	5.141	0.833	5.236 ± 2.969	2.991	0.009 **	0.725
		R	4.953	0.802				
	LLs	L	12.876	1.746	3.236 ± 2.766	1.222	0.239	0.296
		R	12.724	1.704				
%FM	ULs	L	17.259	4.889	5.484 ± 4.372	1.580	0.134	0.383
		R	16.782	5.082				
	LLs	L	18.494	4.303	3.345 ± 2.983	0.164	0.872	0.040
		R	18.324	4.333				
FM (kg)	ULs	L	0.841	0.283	9.198 ± 5.839	2.678	0.016 **	0.650
		R	0.785	0.262				
	LLs	L	2.269	0.689	5.860 ± 2.833	1.590	0.131	0.386
		R	2.215	0.657				
FFM (kg)	ULs	L	4.052	0.725	5.107 ± 2.804	2.449	0.026 *	0.594
		R	3.929	0.717				
	LLs	L	9.989	1.468	3.317 ± 2.848	1.081	0.296	0.262
		R	9.881	1.446				
BMC (kg)	ULs	L	0.249	0.043	4.605 ± 3.194	4.094	<0.001 ***	0.993
		R	0.240	0.043				
	LLs	L	0.610	0.086	2.780 ± 3.352	0.164	0.872	0.040
		R	0.609	0.091				

SD: standard deviation. BC-ASs: body composition asymmetries. *p*: statistical significance value (* *p* < 0.05, ** *p* < 0.01, *** *p* < 0.001). BC: body composition. LIMBS: limbs (upper limbs—ULs—upper body; lower limbs—LLs—lower body). Side (L: left; R: right). ES: effect size expressed as Cohen’s d. TM: Total mass. %FM: fat mass percentage. FM (kg): fat mass. FFM: fat-free mass. BMC: bone mineral content.

**Table 2:** Absolute inter-limb differences according to fighting style.

BC Variables	Limbs	RIGHT	SD	LEFT	SD	*t*	*p*	95% CI Lower	95% CI Upper
GUARD FIGHTERS (n = 10)									
TM (kg)	ULs	4.957	0.665	4.857	0.648	0.810	0.449	−0.202	0.402
	LLs	12.586	1.551	12.500	1.493	0.318	0.761	−0.574	0.745
%FM	ULs	17.100	3.237	16.614	3.154	0.860	0.423	−0.896	1.867
	LLs	18.400	2.346	18.543	2.472	−0.406	0.699	−1.004	0.718
FM (kg)	ULs	0.811	0.191	0.766	0.158	1.009	0.352	−0.064	0.153
	LLs	2.200	0.338	2.195	0.348	0.066	0.950	−0.171	0.180
FFM (kg)	ULs	3.928	0.558	3.870	0.581	0.610	0.564	−0.175	0.291
	LLs	9.796	1.320	9.663	1.259	0.618	0.559	−0.393	0.658
BMC (kg)	ULs	0.233	0.023	0.229	0.026	1.020	0.347	−0.006	0.014
	LLs	0.579	0.060	0.589	0.074	−1.117	0.307	−0.033	0.012
PASS FIGHTERS									
TM (kg)	ULs	5.270	0.945	5.020	0.922	4.038	0.003 **	0.110	0.390
	LLs	13.080	1.924	12.880	1.900	1.762	0.112	−0.057	0.457
%FM	ULs	17.370	5.956	16.900	6.265	1.324	0.218	−0.333	1.273
	LLs	18.560	5.407	18.170	5.407	2.169	0.058	−0.017	0.797
FM (kg)	ULs	0.862	0.342	0.799	0.324	3.287	0.009 **	0.020	0.106
	LLs	2.318	0.872	2.230	0.828	3.212	0.011 *	0.026	0.151
FFM (kg)	ULs	4.138	0.841	3.970	0.827	3.163	0.011 **	0.048	0.289
	LLs	10.124	1.619	10.033	1.611	0.977	0.354	−0.120	0.302
BMC (kg)	ULs	0.260	0.051	0.247	0.052	6.059	<0.001 ***	0.008	0.018
	LLs	0.632	0.097	0.623	0.103	1.101	0.300	−0.010	0.028

Differences in body limbs in terms of body composition between BJJ fighting styles. Definitions: *: *p* < 0.05, **: *p* < 0.005; ***: *p* < 0.001; *t* = t value; *p* = *p* value. Definitions: ULs: upper limbs. LLs: lower limbs. TM: total mass. %FM: percentage of fat mass. FM: fat mass. FFM: fat-free mass. BMC: bone mineral content.

**Table 3:** Comparison of body composition asymmetries according to BJJ fighting style (n = 17).

BC Variables	Limbs	BJJ Style	AS% Mean	SD	*t*	*p*	Mean Difference	Lower	Upper	ES
TM	ULs	Guard Fighters	5.339	3.494	0.116	0.909	0.175	−3.045	3.396	0.057
		Pass Fighters	5.164	2.744						
	LLs	Guard Fighters	4.262	3.361	1.307	0.211	1.744	−1.099	4.587	0.644
		Pass Fighters	2.518	2.163						
%FM	ULs	Guard Fighters	5.965	4.563	0.368	0.718	0.816	−3.906	5.538	0.181
		Pass Fighters	5.149	4.450						
	LLs	Guard Fighters	3.961	3.171	0.700	0.495	1.046	−2.139	4.232	0.345
		Pass Fighters	2.915	2.937						
FM	ULs	Guard Fighters	9.974	7.673	0.447	0.661	1.319	−4.974	7.612	0.220
		Pass Fighters	8.655	4.536						
	LLs	Guard Fighters	7.434	2.851	2.113	0.052	2.675	−0.024	5.373	1.041
		Pass Fighters	4.759	2.363						
FFM	ULs	Guard Fighters	5.141	3.124	0.116	0.909	0.175	−3.045	3.396	0.057
		Pass Fighters	5.084	2.734						
	LLs	Guard Fighters	4.771	3.163	1.307	0.211	1.744	−1.099	4.587	0.644
		Pass Fighters	2.300	2.227						
BMC	ULs	Guard Fighters	3.674	3.178	−1.006	0.330	−1.583	−4.937	1.771	−0.496
		Pass Fighters	5.257	3.203						
	LLs	Guard Fighters	3.108	2.452	0.328	0.748	0.558	−3.067	4.182	0.162
		Pass Fighters	2.550	3.979						

t: Student’s *t*-test value. *p*: statistical significance value. Difference: mean difference between groups. CI: confidence interval for mean difference. ES: effect size expressed as Cohen’s d. Definitions: ULs: upper limbs. LLs: lower limbs. TM: total mass. %FM: percentage of fat mass. FM: fat mass. FFM: fat-free mass. BMC: bone mineral content.

## Data Availability

Data may be requested from the corresponding author.
